# Establishment and health management application of a prediction model for high-risk complication combination of type 2 diabetes mellitus based on data mining

**DOI:** 10.1371/journal.pone.0289749

**Published:** 2023-08-08

**Authors:** Xin Luo, Jijia Sun, Hong Pan, Dian Zhou, Ping Huang, Jingjing Tang, Rong Shi, Hong Ye, Ying Zhao, An Zhang

**Affiliations:** 1 Department of Health Management, School of Public Health, Shanghai University of Traditional Chinese Medicine, Shanghai, China; 2 Department of Mathematics and Physics, School of Pharmacy, Shanghai University of Traditional Chinese Medicine, Shanghai, China; National Healthcare Group, SINGAPORE

## Abstract

In recent years, the prevalence of T2DM has been increasing annually, in particular, the personal and socioeconomic burden caused by multiple complications has become increasingly serious. This study aimed to screen out the high-risk complication combination of T2DM through various data mining methods, establish and evaluate a risk prediction model of the complication combination in patients with T2DM. Questionnaire surveys, physical examinations, and biochemical tests were conducted on 4,937 patients with T2DM, and 810 cases of sample data with complications were retained. The high-risk complication combination was screened by association rules based on the Apriori algorithm. Risk factors were screened using the LASSO regression model, random forest model, and support vector machine. A risk prediction model was established using logistic regression analysis, and a dynamic nomogram was constructed. Receiver operating characteristic (ROC) curves, harrell’s concordance index (C-Index), calibration curves, decision curve analysis (DCA), and internal validation were used to evaluate the differentiation, calibration, and clinical applicability of the models. This study found that patients with T2DM had a high-risk combination of lower extremity vasculopathy, diabetic foot, and diabetic retinopathy. Based on this, body mass index, diastolic blood pressure, total cholesterol, triglyceride, 2-hour postprandial blood glucose and blood urea nitrogen levels were screened and used for the modeling analysis. The area under the ROC curves of the internal and external validations were 0.768 (95% CI, 0.744−0.792) and 0.745 (95% CI, 0.669−0.820), respectively, and the C-index and AUC value were consistent. The calibration plots showed good calibration, and the risk threshold for DCA was 30–54%. In this study, we developed and evaluated a predictive model for the development of a high-risk complication combination while uncovering the pattern of complications in patients with T2DM. This model has a practical guiding effect on the health management of patients with T2DM in community settings.

## Introduction

Diabetes mellitus (DM), a common chronic noncommunicable disease, is a metabolic disorder caused by dysfunction in the secretion or utilization of insulin in the human body. With the improvement in the living standard of the population, its prevalence is increasing, and DM is a major global health problem [1]. According to a report by the International Diabetes Federation, 537 million adults (aged 20–79 years) worldwide will have DM in 2021, with one in 10 people living with DM, and this number is expected to increase to 783 million by 2045. Global health spending on DM amounts to $966 billion and is expected to reach $1054 billion by 2045. Currently, DM is the ninth leading cause of death in humans, and complications from persistent hyperglycemia are an important cause of death and disability in patients, with more than four million adults dying from DM complications [2, 3]. The World Health Organization reports more than 100 complications of DM, including eye, nerve, kidney, and cardiovascular diseases and pregnancy and oral complications. More than 90% of all patients with DM have type 2 DM (T2DM) [4], and Asia is the main region where the rapid prevalence of T2DM occurs, with China, India, and Pakistan topping the list of T2DM prevalence. A Lancet study predicts global age-standardized prevalence of T2DM to increase by more than 60% by 2050 [5], and the direct or indirect annual per capita cost of treating T2DM ranges from $29.91 to $237.38 [6].

Currently, there is no complete treatment for T2DM, relying mainly on existing medications and a healthy lifestyle to control the development of the disease [7]. However, more than 50% of patients with T2DM are unaware of their disease [8], leading to the development or exacerbation of complications in various parts of the body and reducing the quality of life. Therefore, based on the screening of patients with T2DM, there is an urgent need for better medical tools and interventions to prevent and stop the development of complications.

Clinical prediction models are mathematical models that use parameters (nonparametric) to predict the probability and likelihood of an outcome event occurring in the present or future of a study population. As a quantitative tool for assessing risks and benefits, it provides healthcare professionals with visual and accurate data information, and its use is becoming increasingly common [9, 10]. Through a literature review, we found that most of the studies that established prediction models for T2DM in the Chinese region were single predictions of T2DM or some type of complications [11–15], and few studies have been reported on the pattern and prediction of T2DM complications. Machine learning(ML), a branch of artificial intelligence, plays a crucial role in various applications by employing diverse algorithms and statistical models to enable computer systems to learn from data and extract meaningful patterns. This capability empowers ML to make predictions and informed decisions across a wide range of fields, including computer vision, image processing technology and healthcare, etc. [16–18]. Particularly in the realm of clinical predictive models, ML provides powerful tools and methods for their development and application.

In China, with the aggravation of the social disease burden caused by chronic diseases and the implementation of hierarchical medical systems, the management focus of chronic diseases, such as diabetes, has gradually shifted to primary medical institutions [19]. Community health workers play an important role in T2DM management. Strategies on how to further improve and strengthen the management of chronic diseases, such as T2DM, in the community, remain a current study hotspot [20–23].

In this study, based on the collected T2DM community dataset, association rule analysis based on the Apriori algorithm was used to identify the high-risk complication combination of T2DM, develop a risk prediction model, and explore the application of a prediction model in community chronic disease management.

## Materials and methods

### Sample collection and data processing

This was a retrospective study. The sample data were obtained from the Zhangjiang area in the Pudong New Area of Shanghai, led by the School of Public Health of Shanghai University of Traditional Chinese Medicine. Moreover, with the assistance of Huamu, Jinyang, Yinxing, Siping, Sanlin, and Daqiao community health service centers in Pudong New Area, Shanghai, the project of “The fourth round of three-year action plan for public health construction of key disciplines of health education and promotion” was completed. Through questionnaire surveys and physical, biochemical, fundus, and peripheral nerve examinations, the project collected relevant data from community residents who participated in the community T2DM cohort project in six community health service centers from December 2015 to April 2016. The study has been ethically approved by the medical ethics committee of Longhua Hospital, Shanghai University of Traditional Chinese Medicine and in accordance with the Declaration of Helsinki ethical principles and guidelines. Written informed consent of all participants were obtained after they were briefed about the study’s objectives. According to the principle of matching age and sex, randomization, and no blood relationship among all study subjects, the research group obtained data from 4937 patients with T2DM and 86 indicators in the baseline information collection stage.

According to the purpose of the study, the study firstly excluded 56 irrelevant indicators, such as patients’ dietary management, exercise management and community management, and retained the occurrence of complications, basic demographic indicators and physical examination indicators in T2DM patients, a total of 29 indicators, which involved a total of 10 diabetic complications and 19 basic variables, including diabetic ketoacidosis, diabetic nephropathy, diabetic cardio-cerebrovascular complications, hyperglycemic hyperosmolar state, diabetic retinopathy, lower extremity vascular disease, lactic acidosis, diabetic neuropathy, diabetic foot, hypoglycemia, family history of DM, gender, waist-to-hip ratio (WHR), age, body mass index (BMI), course of DM, systolic blood pressure (SBP), diastolic blood pressure (DBP), glucose (GLU), 2-hour postprandial blood glucose (GLU2H), glycosylated hemoglobin-Type A1C (HbA1c), total cholesterol (TC), triglyceride (TG), high-density lipoprotein (HDL), blood urea nitrogen (BUN), serum creatinine (SCR), uric acid (UA), urinary creatinine (UCR), and urinary microalbumin (UMA). Correlation analysis of 19 basic variables was performed using the corrplot package (version 0.8.24).

Secondly, the study only retained data on 810 cases suffering from related complications, of which a total of 138 data were missing (17%). Considering the impact of missing data on the results, the study processed the data by multiple interpolation through IBM SPSS statistics (version 25.0). The study used automatic interpolation method by default, i.e., the interpolation method was selected automatically based on the data scanning results. 5 interpolations were performed to obtain 5 datasets without missing values, and the 5 datasets were compared with the original data by reliability analysis, and the dataset with the largest Cronbach’s α value (higher value indicates higher consistency within the data) was selected as the base data for the subsequent study. The data of this study are shown in S1 File.

Finally, data analysis of the final sample of 810 cases included in this study was performed using IBM SPSS software, including basic statistical descriptions under the grouping for whether the complication combination occurred, and the chi-squared and one-sample nonparametric tests were used to analyze whether the differences between groups were statistically significant. All statistical tests used were two-sided, with a test level of 0.05.

### Statistical and modeling methods

Association rule analysis primarily refers to the process of discovering potential relationships and rules between study subjects in large-scale datasets. It is widely used in medicine and economics, and the most classic algorithm is the Apriori algorithm, which can determine the frequent item set and importance in the dataset according to some indicators, such as support, confidence, and lift [24, 25]. Support indicates the proportion of rules that occur, confidence indicates the reliability of the rules, and lift indicates whether there is significance between the rules, with a value equal to 1 being no association and greater than 1 being a positive correlation. Therefore, this study used arules (version 1.7–3), arulesViz (version 1.5–1), tidyverse (version 1.3.1), kableExtra (version 1.3.4), and other packages of R software(version 4.1.0, https://www.R-project.org/) to mine association rules for the included T2DM complication dataset and to determine the high-risk complication combination. The risk prediction model was established by using the “whether patients with T2DM have concurrent complication combination” as the outcome variable.

The least absolute shrinkage and selection operator (LASSO) regression analysis mode based on a 10-fold cross-validation, random forest (RF) model, and support vector machine (SVM) was used to screen the risk factors of this high-risk complication combination in patients with T2DM. The common variables selected by the three methods were incorporated into the prediction model. The LASSO regression model is a variable selection method of the linear regression model, which is suitable for large datasets. We used the glmnet package (version 4.1–2) in R software to centralize and normalize the variables included in the analysis so that the coefficients of the variables shrank to zero and screened out the prediction variables with non-zero coefficients under the lambda value of the best penalty parameter under the condition of minimizing the prediction error [15, 26, 27]. RF is a machine learning method that can be used for classification and prediction and is applicable to all types of data. To avoid the overfitting problem, the prediction variables are measured and identified in the form of importance ranking [28, 29], which is completed by the randomForest (version 4.6–14) and varSelRF (version 0.7–8) packages in R software. SVM is a powerful class of generalized linear classifiers that classifies data in supervised learning and compared with other machine learning methods, it has more advantages in identifying subtle patterns in large-scale datasets [30]. A study review also proposed the validity of the SVM in T2DM studies [31]. Therefore, this study used an SVM based on a linear kernel function to screen predictive variables, which was completed by the e1071 (version 1.7–9), kernlab (version 0.9–29), and caret (version 6.0–88) packages in R software.

The outcome variable of this study was the dichotomous variable “whether patients with T2DM have concurrent complication combination.” Therefore, logistic regression analysis was used to construct a prediction model containing the final variables. When the screened common variables were included in the multifactor logistic regression analysis, the predictor variables with P > 0.05 were excluded so that all predictor variables included in the model were statistically significant. The rms (version 6.2–0), DynNom (version 5.0.1), and rsconnect (version 0.8.24) packages in R software were used to draw the nomogram and the online web application of the prediction model. The length of the line corresponding to each variable in the column line graph was proportional to the degree of influence of that variable on the predicted outcome [32, 33]. A nomogram can predict the possibility of disease occurrence simply and effectively and can serve as a clinical decision support tool and reduce healthcare costs [34].

Finally, multiple tests were used in the training and validation sets to assess the discrimination, calibration, and clinical application values of the prediction model. The receiver operating characteristic (ROC) curve and the area under the ROC curve (AUC) were used to assess whether the differentiation ability of the model met the requirements [35], and the closer the AUC value is to 1, the better the differentiation ability of the model [27]. A calibration curve was used to demonstrate the calibration capability of the model and assess the degree of agreement between the actual situation and the predicted results of the model. While AUC and calibration curves provide measures of model accuracy and predictive performance, they do not consider patient risk thresholds and decision consequences. In contrast, Decision Curve Analysis (DCA) can incorporate these factors to comprehensively assess and compare the utility and clinical value of predictive models, so the net benefit of the clinical decision scenarios based on this predictive model was supplemented using decision DCA [36, 37].

**Fig 1:**
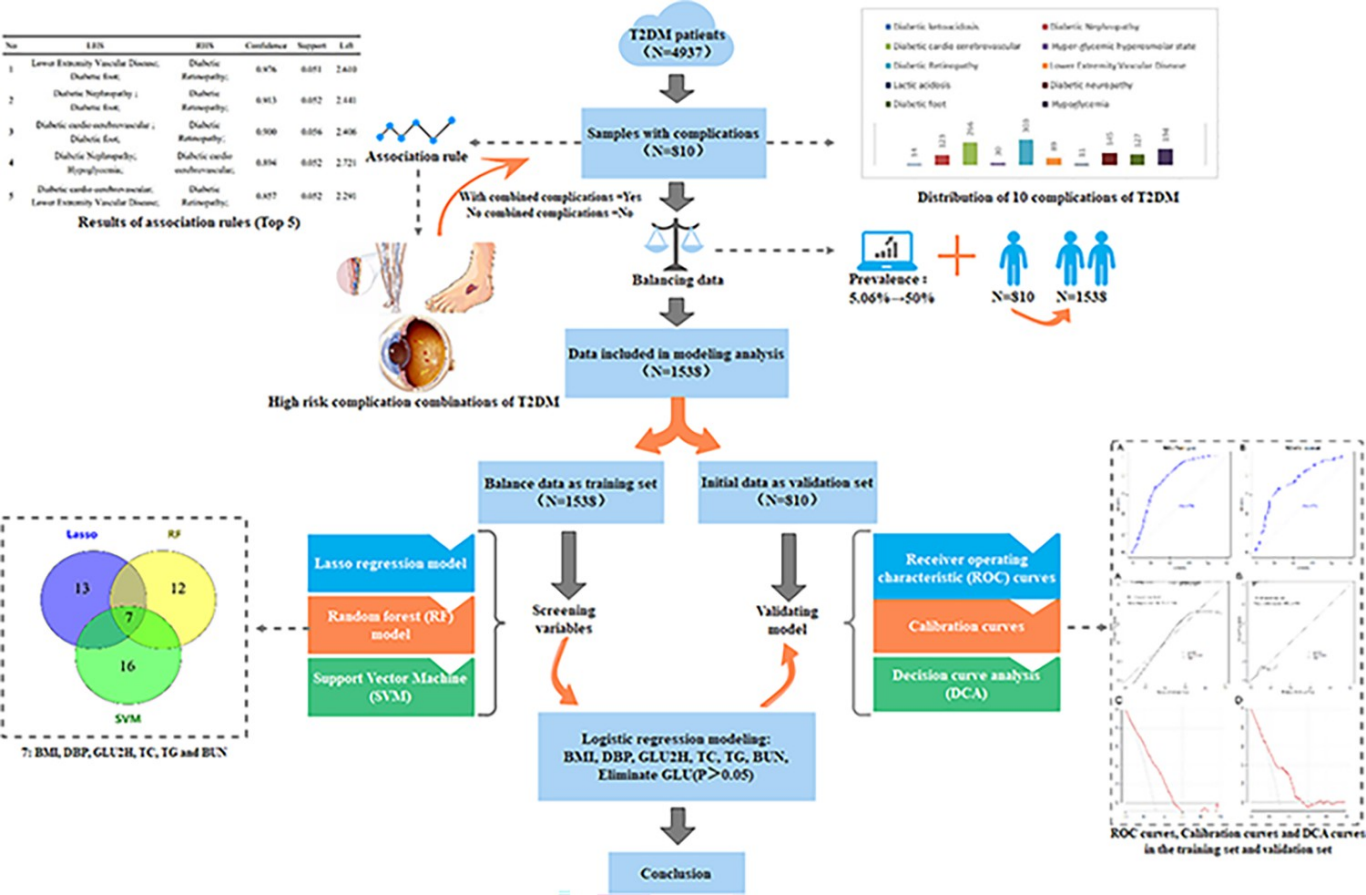
Flow diagram of the study design.

The flowchart of the study design is shown in Fig 1.

## Results

### Association rule analysis

After preprocessing, the data from the 810 sample cases were analyzed in two steps. The data containing complication conditions were first analyzed by association rules based on the Apriori algorithm, and the combination of whether or not a complication occurred (yes = 1, no = 0) was reincorporated into the sample data for modeling analysis based on identifying high-risk complication combinations.

**Fig 2:**
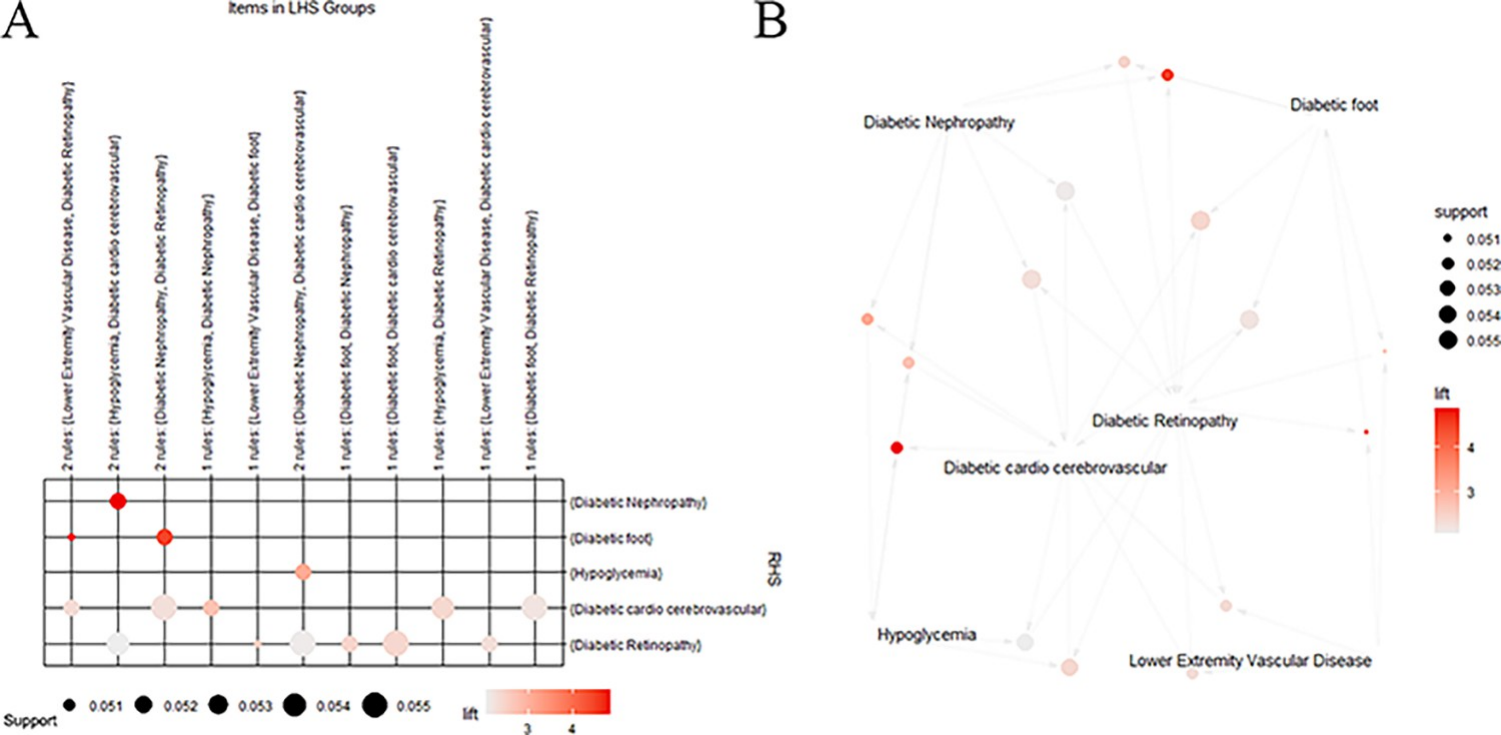
Association rule analysis using Apriori-based algorithm. (A) Grouping matrix graph of 15 association rules mined by the algorithm. (B) Network relationship diagram of 15 association rules.

The R software was used to mine association rules based on the Apriori algorithm for sample data containing complications. Setting the minimum confidence level, minimum support level, and minimum lift to 0.07, 0.05, and 1, respectively, 15 frequent itemset were found (Fig 2). The results were sorted according to the magnitude of the confidence level and are shown in Table 1. The highest-confidence complication combinations (lower extremity vascular disease, diabetic foot, and diabetic retinopathy) were selected, and the complication combination was used as an outcome variable for modeling analysis.

**Table 1:** Apriori algorithm-based association rules.

No	LHS	RHS	Confidence	Support	Lift
1	Lower Extremity Vascular Disease; Diabetic foot;	Diabetic Retinopathy;	0.976	0.051	2.610
2	Diabetic Nephropathy; Diabetic foot;	Diabetic Retinopathy;	0.913	0.052	2.441
3	Diabetic cardio cerebrovascular; Diabetic foot;	Diabetic Retinopathy;	0.900	0.056	2.406
4	Diabetic Nephropathy; Hypoglycemia;	Diabetic cardio cerebrovascular;	0.894	0.052	2.721
5	Diabetic cardio cerebrovascular; Lower Extremity Vascular Disease;	Diabetic Retinopathy;	0.857	0.052	2.291
6	Diabetic Nephropathy; Diabetic cardio cerebrovascular;	Diabetic Retinopathy;	0.789	0.056	2.110
7	Diabetic Retinopathy; Hypoglycemia;	Diabetic cardio cerebrovascular;	0.786	0.054	2.393
8	Diabetic cardio cerebrovascular; Hypoglycemia;	Diabetic Retinopathy;	0.772	0.054	2.064
9	Diabetic Retinopathy; Lower Extremity Vascular Disease;	Diabetic cardio cerebrovascular;	0.764	0.052	2.325
10	Diabetic Nephropathy; Diabetic Retinopathy;	Diabetic cardio cerebrovascular;	0.750	0.056	2.284
11	Diabetic Retinopathy; Lower Extremity Vascular Disease;	Diabetic foot;	0.745	0.051	4.754
12	Diabetic Nephropathy; Diabetic cardio cerebrovascular;	Hypoglycemia;	0.737	0.052	3.077
13	Diabetic cardio cerebrovascular; Hypoglycemia;	Diabetic Nephropathy;	0.737	0.052	4.852
14	Diabetic Retinopathy; Diabetic foot;	Diabetic cardio cerebrovascular;	0.726	0.056	2.210
15	Diabetic Nephropathy; Diabetic Retinopathy;	Diabetic foot;	0.700	0.052	4.465

Note: The magnitude of the confidence level in the table indicates the prevalence of the complication combination.

### Basic statistical description

**Fig 3:**
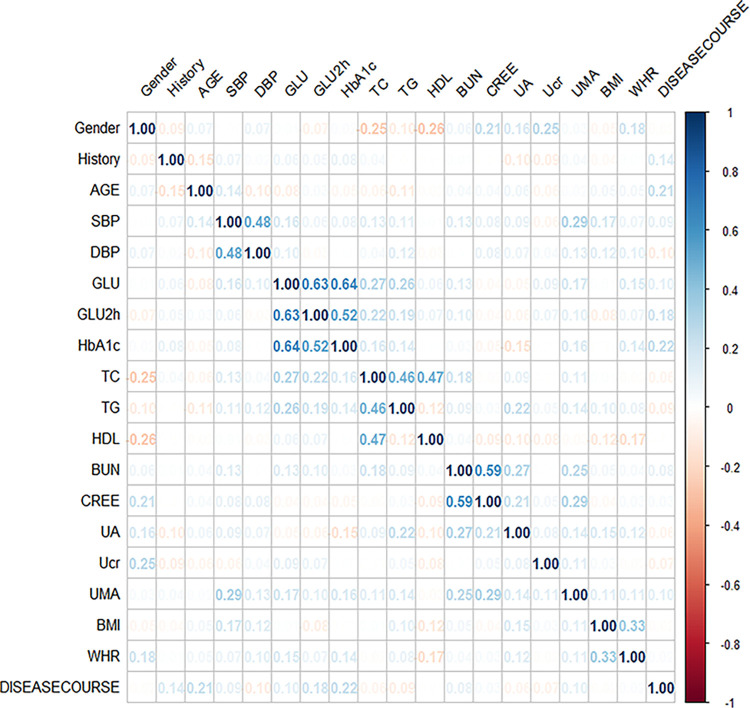
Correlation analysis of 19 variables. Blue indicates positive correlation, red indicates negative correlation.

**Fig 4:**
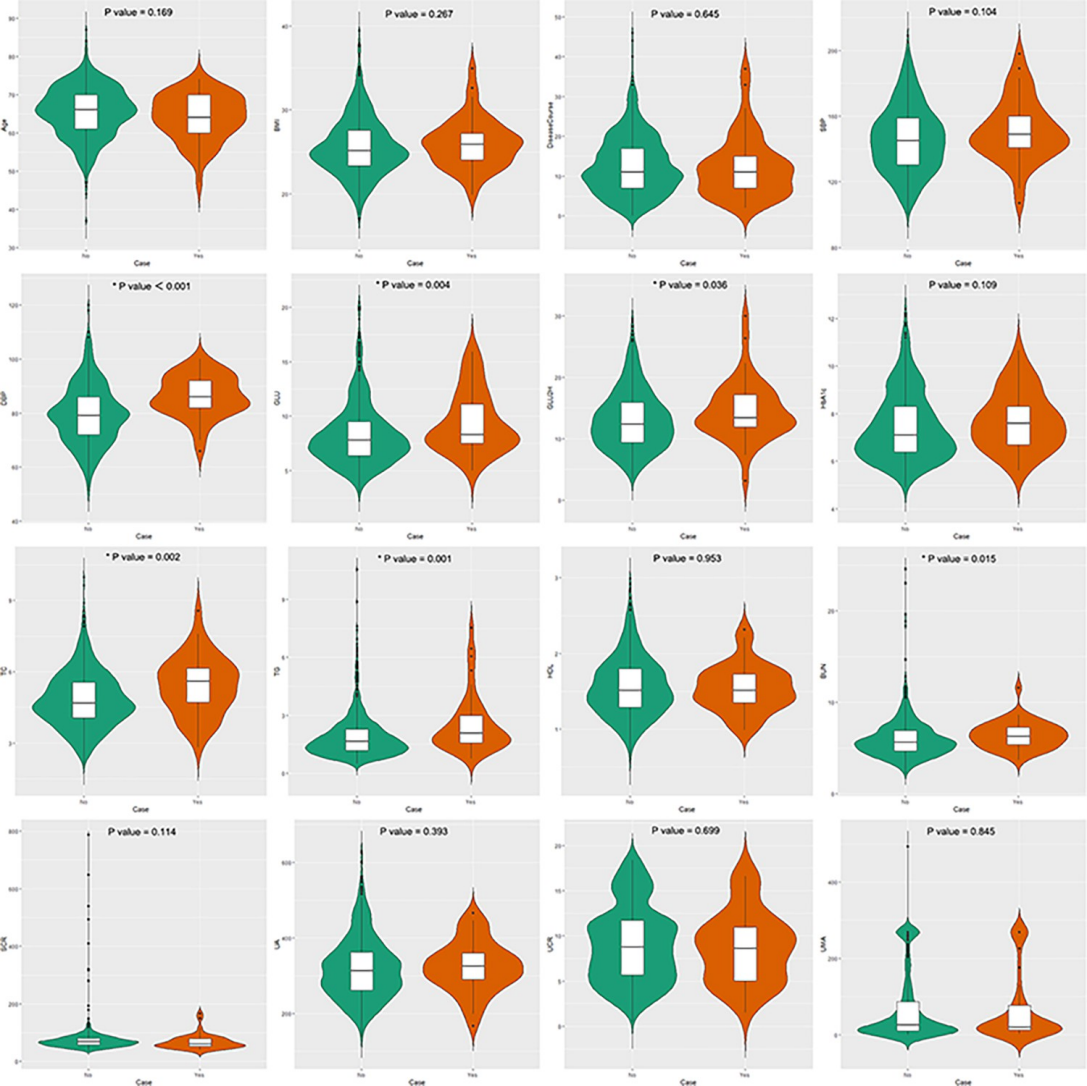
Violin plot of 16 clinical variables. It shows the differences between groups and comparative analysis for the condition " whether patients with T2DM have concurrent the complication combination ".

The correlation analysis of 19 variables is shown in Fig 3 and the basic statistical description under the grouping for whether the complication combination occurred is shown in Table 2 and Fig 4.

**Table 2:** Differences in demographic and clinical characteristics between the no case and case groups. [n(%)/median(IQR)].

Demographic characteristics	Case				χ2/Z	P	Total (n = 810)
	No		Yes				
**Family history of DM**							
No	420 (54.6)		17 (41.5)		2.711	0.100	437 (54.0)
Yes	349 (45.4)		24 (58.5)				373 (46.0)
**Gender**							
Male	358 (46.6)		19 (46.3)		0.001	0.979	377 (46.5)
Female	411 (53.4)		22 (53.7)				433 (53.5)
**WHR**							
Normal	238 (30.9)		8 (19.5)		2.408	0.121	246 (30.4)
Obesity	531 (69.1)		33 (80.5)				564 (69.6)
**Age (years)**	66.00 (9.00)		64.00 (10.00)		1.375	0.169	
**BMI (kg/m** ^**2**^ **)**	25.11 (4.20)		25.91 (3.40)		1.110	0.267	
**Course of DM**	11.00 (10.00)		11.00 (8.50)		0.461	0.645	
**SBP (mmHg)**	145.00 (29.00)		149.00 (22.00)		1.624	0.104	
**DBP (mmHg)**	79.00 (15.00)		86.00 (11.00)		4.643	<0.001*	
**Glu (mmol/L)**	7.80 (3.10)		8.30 (3.75)		2.892	0.004*	
**Glu2h (mmol/L)**	12.40 (6.50)		13.40 (5.45)		2.095	0.036*	
**HbA1c (%)**	7.10 (1.90)		7.60 (1.85)		1.602	0.109	
**TC (mmol/L)**	4.68 (1.51)		5.59 (1.54)		3.171	0.002*	
**TG (mmol/L)**	1.64 (1.11)		2.07 (1.43)		3.285	0.001*	
**HDL (mmol/L)**	1.51 (0.51)		1.51 (0.40)		0.059	0.953	
**BUN (mmol/L)**	5.63 (2.28)		6.27 (2.05)		2.436	0.015*	
**SCR (mmol/L)**	68.00 (24.00)		61.00 (29.00)		1.581	0.114	
**UA (μmol/L)**	313.00 (101.00)		325.00 (74.50)		0.855	0.393	
**UCR (μmol/L)**	8.75 (6.10)		8.61 (6.02)		0.387	0.699	
**UMA (mg/L)**	26.00 (76.00)		20.00 (69.50)		0.196	0.845	

### Screening results of characteristic variables

In the sample data of 810 patients in this study, 41 (5.06%) experienced a combination of complications, with relatively unbalanced positive and negative sample data. The study dataset was balanced by the Synthetic Minority Oversampling Technique in SPSSPRO software (version 1.1.1), and the balanced dataset consisted of 1538 cases, including 769 cases with the complication combination (50.0%). To ensure the accuracy and validity of the model-building results, this study used a balanced dataset (n = 1538) as the training set and the original dataset before balancing (n = 810) as the validation set.

**Fig 5:**
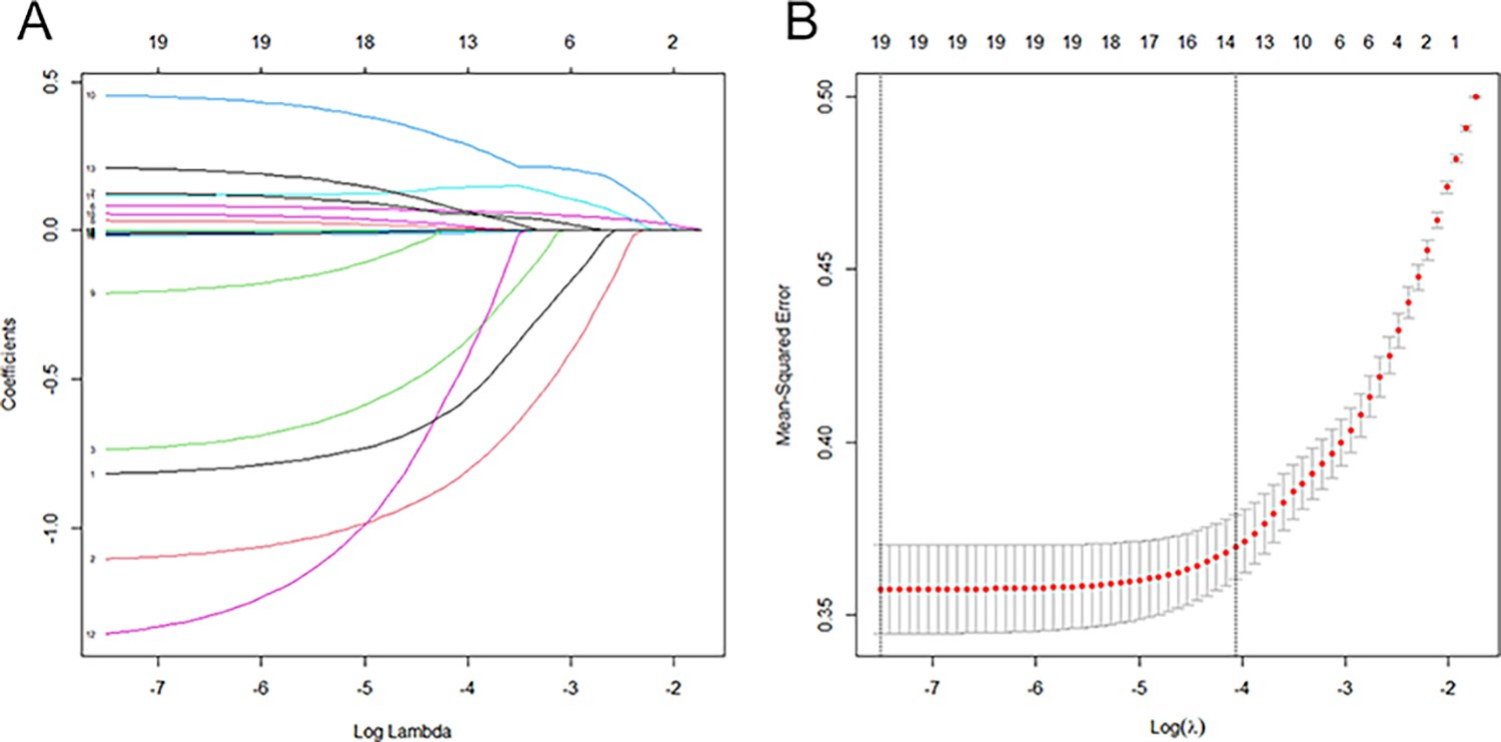
Variable screening using the LASSO regression model. (A) Determination of the best penalty coefficient lambda value. (B) Coefficient profiles according to the best parameter (lambda) in Fig 5(A).

**Fig 6:**
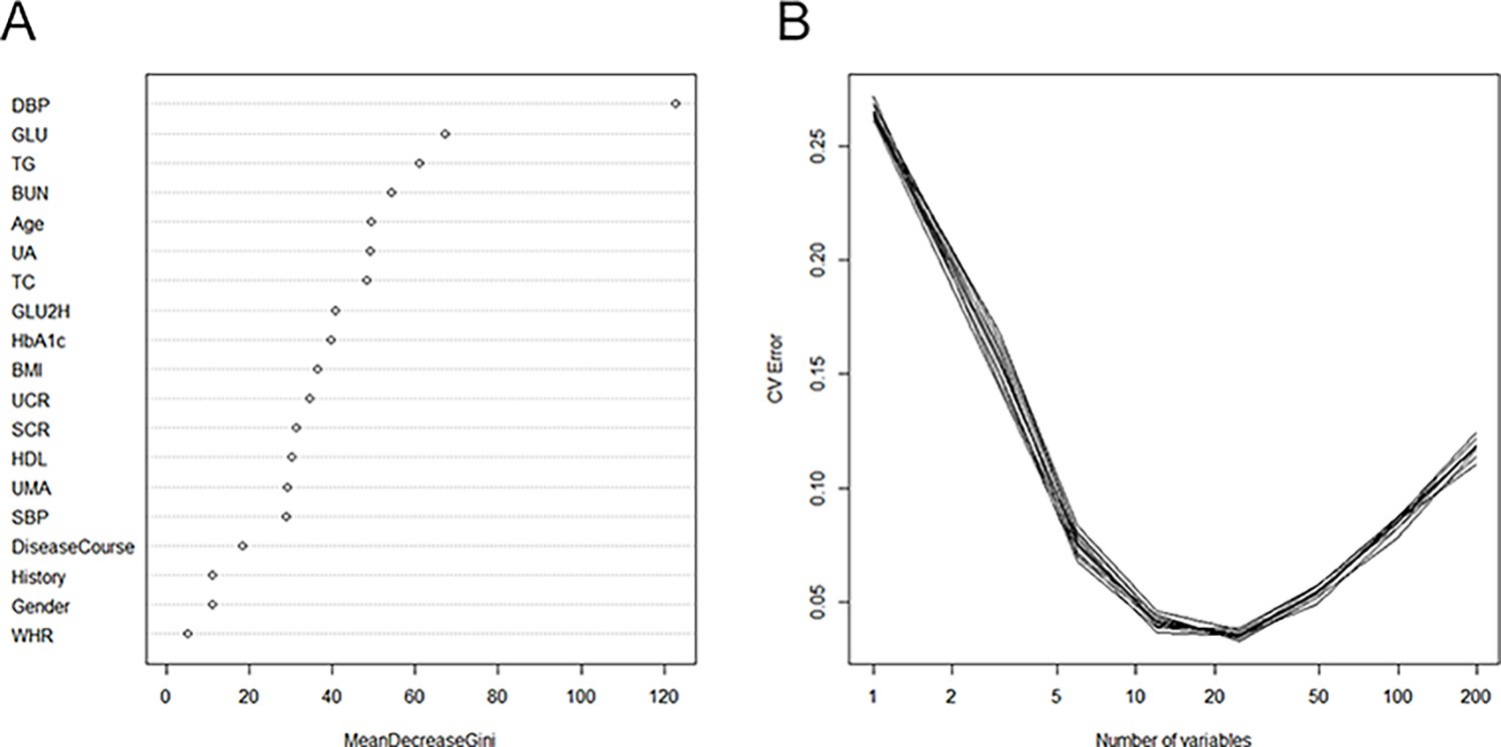
Importance ranking and 10-fold cross-validation of the analyzed variables using a random forest model to determine the optimal number of variables. (A) Importance ranking of the included variables. (B) Ten-fold cross-validation of the random forest model, which showed the model error rate is the lowest when the number of variables is 12 among 19 variables.

**Fig 7:**
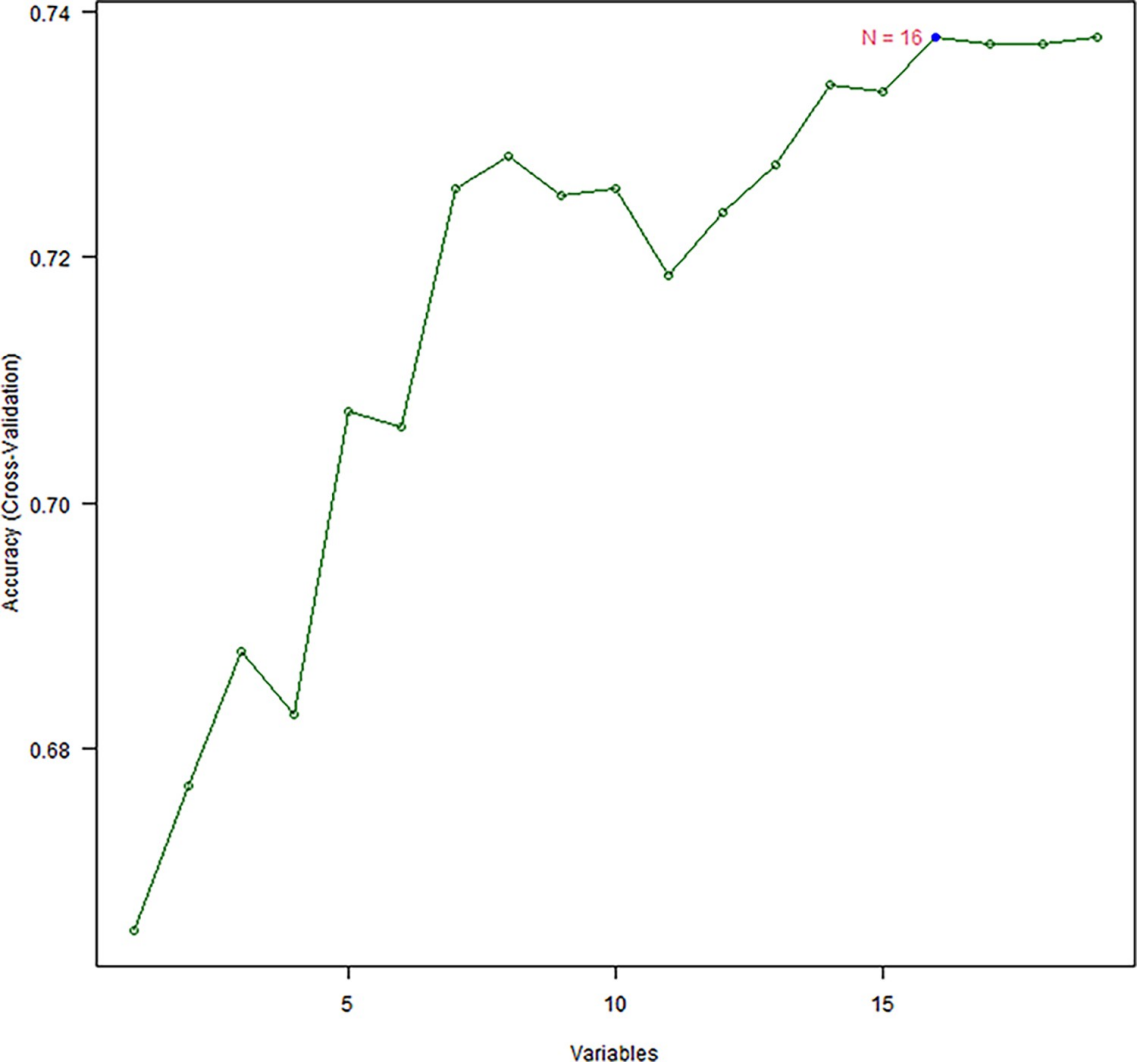
The support vector machine based on linear kernel function has the highest model accuracy when the number of variables is 16.

Variables were screened by LASSO regression analysis, RF model, and SVM in the training set. Among the 19 underlying variables, 13 characteristic variables were screened based on a 10-fold cross-validated LASSO regression analysis with the best lambda of 0.017159 (Fig 5). The 19 variables included in the analysis were ranked in the order of importance using the RF model (Number of trees = 134, OOB estimate of error rate = 2.47%), and a 10-fold cross-validation was applied to determine the appropriate number of variables. The results showed that the model had the lowest error rate when the number of variables was 12; therefore, the top 12 variables in terms of importance were selected as the characteristic variables (Fig 6). The results of the SVM variable screening based on linear kernel functions showed that the highest model accuracy was achieved when the number of variables was 16, with accuracy value of 0.738 (Fig 7). The variables filtered by the three methods were intersected to obtain the following common variables: BMI, DBP, GLU, GLU2H, TC, TG and BUN (Table 3).

**Table 3:** Variable screening results of three methods.

Methods	Variables	Common variables
**LASSO (n = 13)**	Gender, History, WHR, Age, BMI, DBP, GLU, GLU2H, TC, TG, HDL, BUN, UMA;	BMI, DBP, GLU, GLU2H, TC, TG, BUN;
**RF (n = 12)**	Age, BMI, DBP, GLU, GLU2H, HbA1c, TC, TG, BUN, SCR, UA, UCR;	
**SVM (n = 16)**	History, Gender, WHR, BMI, Disease Course, SBP, DBP, GLU, GLU2H, HbA1c, TC, TG, HDL,BUN, SCR, UMA;	

### Model construction and prediction results

Using the eight indicators selected above as independent variables and “whether patients with T2DM have concurrent complication combination” as the dependent variable, a model was constructed using logistic regression. Excluding GLU (P > 0.05), a risk prediction model for the complication combination in patients with T2DM was obtained (Table 4).


$$y_{model}=-11.069+0.045\cdot BMI+0.077\cdot DBP+0.358\cdot TC+0.214\cdot TG+0.049\cdot GLU2H+0.085\cdot BUN$$ (1)

**Table 4:** Predictive factors for the incidence risk of complication combination in patients with T2DM.

	β	z-value	P-value	OR	2.5% CI	97.7% CI
Intercept	-11.069	-12.91	<0.001	1.559e-05	2.808e-06	8.109e-05
BMI	0.045	2.26	0.024	1.046	1.006	1.088
DBP	0.077	11.04	<0.001	1.080	1.066	1.096
TC	0.358	5.90	<0.001	1.431	1.272	1.613
TG	0.214	3.79	<0.001	1.238	1.110	1.385
GLU2H	0.049	3.44	<0.001	1.050	1.021	1.079
BUN	0.085	2.45	0.014	1.089	1.017	1.166

**Fig 8:**
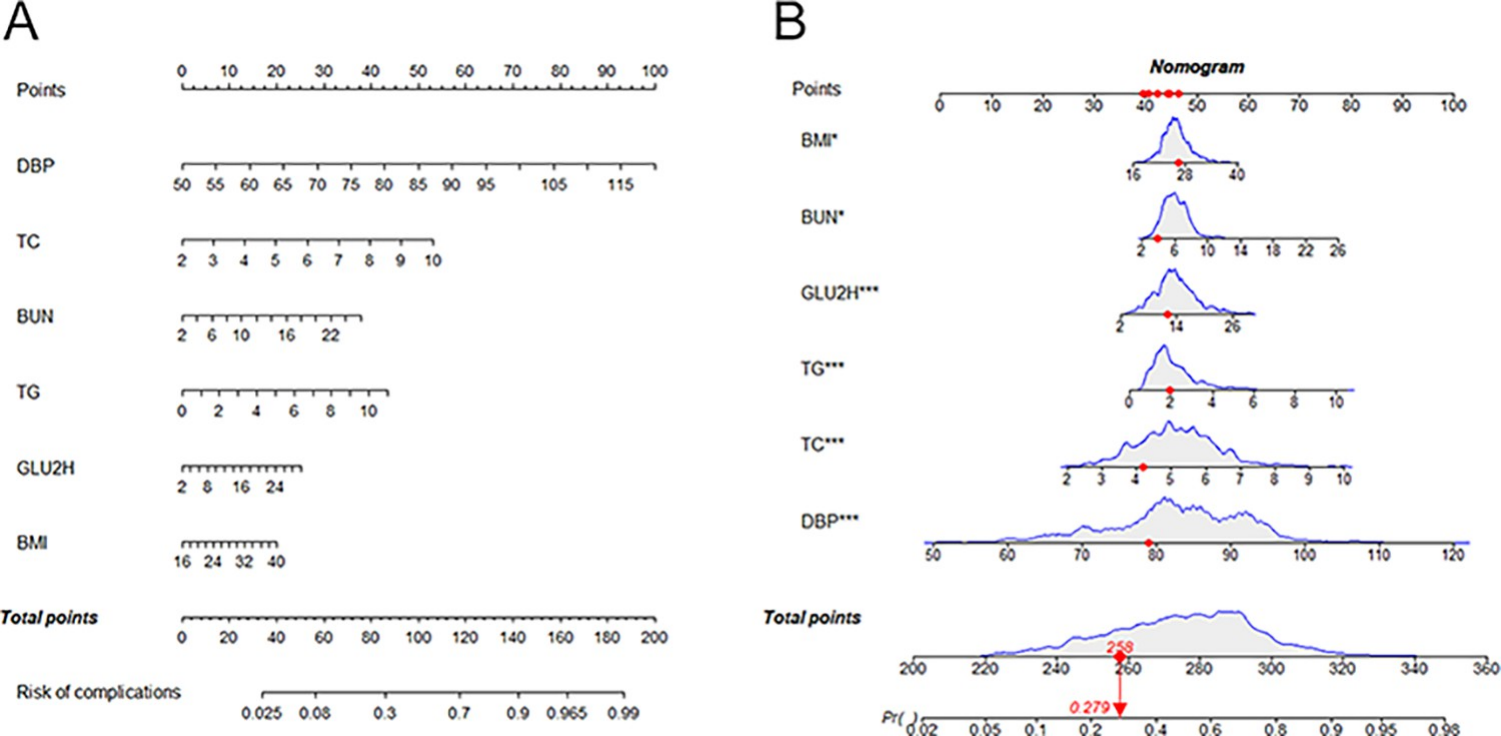
Nomogram of the risk of developing the complication combination in patients with T2DM. (A) Nomogram incorporating 6 variables of BMI, DBP, GLU2H, TC, TG and BUN for analysis. (B) One patient with T2DM was randomly selected from the sample to predict the risk of developing the complication combination based on the 6 characteristic variables of the Nomogram.

The nomogram drawn according to this model could simply and intuitively predict the risk of complications in patients with T2DM (Fig 8). For example, suppose a patient with T2DM has the following characteristics: BMI, 26.37 kg/m^2^; DBP, 79 mmHg; GLU2H, 12.10 mmol/L; TC, 4.18 mmol/L; TG, 1.94 mmol/L; BUN, 3.90 mmol/L; with a score of 258, corresponding to a probability of 0.279, indicating that the risk of concurrent complications in this patient with T2DM is 27.9%. Based on this prediction model, this study made an online application to predict the probability of combined complications in patients with T2DM, which is available at https://studentluo.shinyapps.io/DynNomapp_T2DM/, which can help medical workers better prevent and manage complications in patients with T2DM.

**Fig 9:**
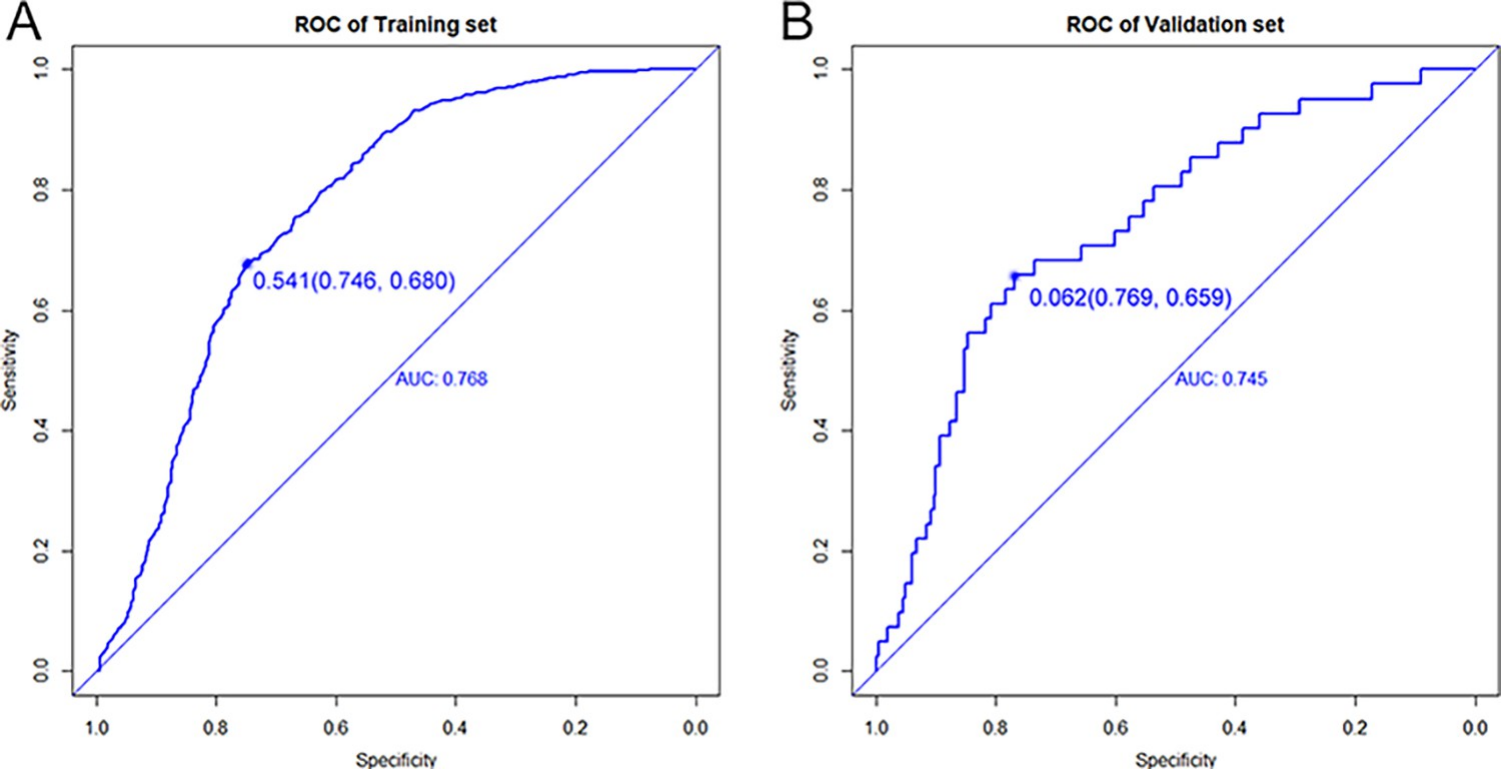
ROC curve analysis. (A) Performance of the model in the training set, which showed an AUC value of 0.768, an optimal cut-off value of 0.541, a specificity of 0.746, and a sensitivity of 0.680. (B) Performance of the model in the validation set, which showed an AUC value of 0.745, an optimal cut-off value of 0.062, a specificity of 0.769, and a sensitivity of 0.659.

**Fig 10:**
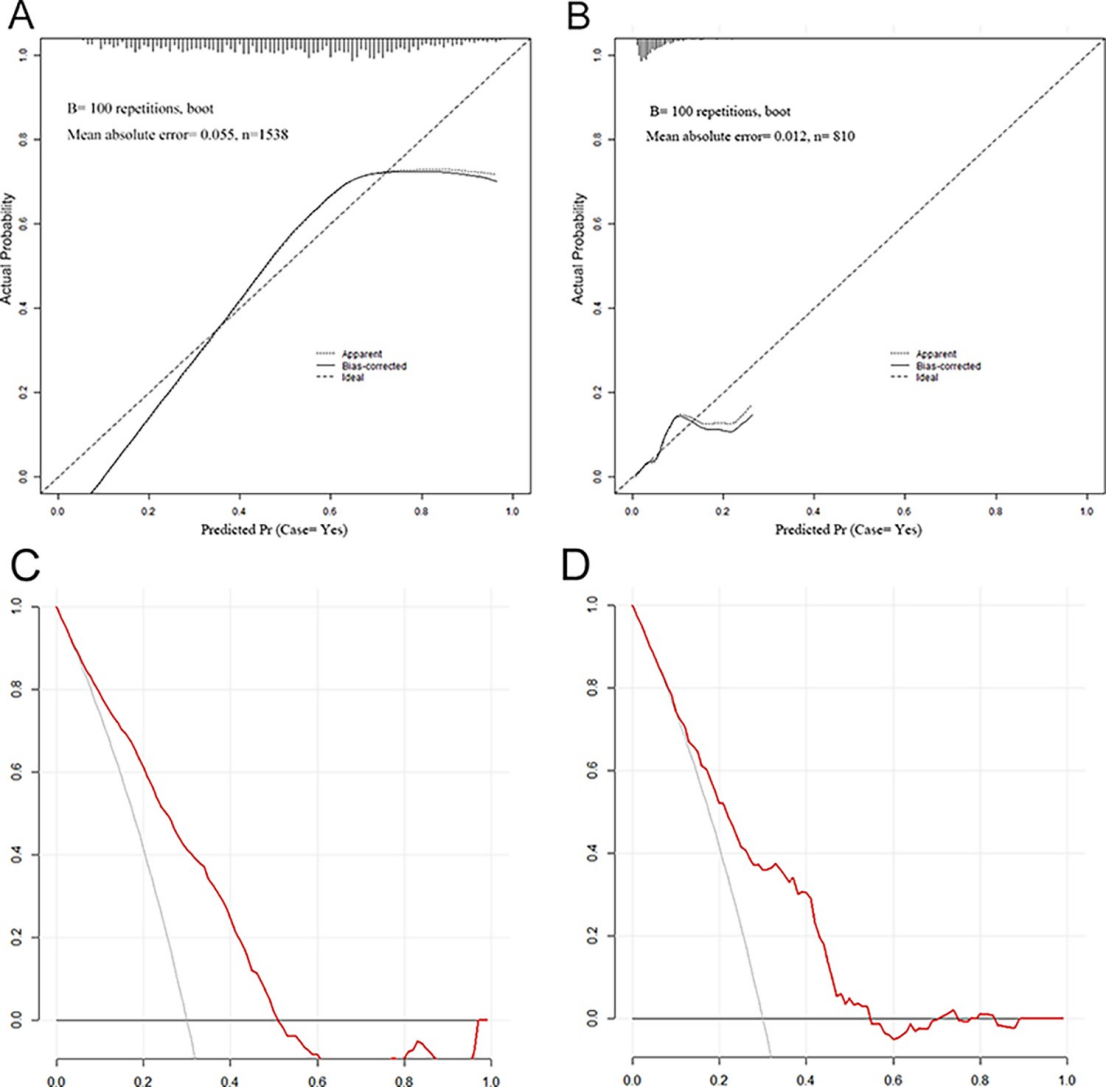
Calibration curve plots and decision curve analysis. (A) Calibration curve plot in the training set, and the solid lines indicate the performance of the model in this study, and diagonal lines indicate perfect predictions of the ideal model. (B) Calibration curve plot in the validation set. (C) Decision curve plot in the training set, when the risk threshold is 30% - 54%, the net benefits from clinical decisions based on this study model. (D) Decision curve plot in the validation set, the risk threshold is 30% - 54%.

The ROC curve is shown in Fig 9, where the AUCs of the training and validation sets were 0.768 (95% confidence interval [CI], 0.744−0.792) and 0.745 (95% CI, 0.669−0.820), respectively, and the C-index and AUC value were consistent. The model had good differentiation ability. The calibration of the model was evaluated using a calibration curve (Fig 10A and 10B), and the results suggested that the model calibration capability (fitting effect) was good. The DCA is shown in (Fig 10C and 10D), and the net benefits of the model in the validation sets was good, suggesting that the model can be applied to clinical decision-making.

## Discussion

### High-risk complication combination of T2DM

Among the common complications of DM, this study found that patients with T2DM had a higher risk of three complication combinations: lower extremity vascular disease, diabetic foot, and diabetic retinopathy. The algorithm results suggest that patients with T2DM have a 97.6% risk of diabetic retinopathy in the presence of lower extremity vascular disease and diabetic foot. Some of the findings suggest a correlation between the three T2DM complications of lower extremity vascular disease, diabetic foot, and diabetic retinopathy, with the three diseases affecting each other [38]. The prognosis and risk factors for the development of diabetic foot in patients with lower extremity vascular disease and diabetic retinopathy as one of the risk factors for lower extremity vascular disease [39] suggest that the results are consistent with the pathological basis for the development of DM complications. In patients with T2DM, lower extremity vascular disease is difficult to detect at an early stage, and as the disease further deteriorates, it will lead to adverse consequences, such as amputation of the patient’s limbs [40]. As a common complication of DM, diabetic foot is a major cause of disability, death, and increased medical burden for patients [41]. Diabetic retinopathy is a common microvascular complication of DM, with 34.6% of patients experiencing both retinopathy and blindness [42], but studies on the diagnosis and treatment of diabetic retinopathy remain inadequate [43]. In summary, the occurrence of the combination of the three T2DM complications identified in this study will result in reduced quality of life, increased medical burden, and increased risk of premature death for patients. Regarding lower extremity vascular disease, diabetic foot, and diabetic retinopathy, currently, the main treatment modality is dependent on early screening and preventive management [44]. Thus, it is necessary to predict the risk of developing the complication combination in patients with T2DM at an early stage and establish targeted intervention measures.

### Risk factor analysis of the complication combinations

This study screened for six risk factors for high-risk joint complications in patients with T2DM: BMI, DBP, TC, TG, GLU2H and BUN. First, the study population is patients with T2DM, and GLU2H is one of the important indicators for discovering and diagnosing diabetes. The development of cardiovascular diseases, such as hypertension evaluated by indicators of SBP and DBP, will increase the risk of lower extremity vascular disease, diabetic foot, and diabetic retinopathy [39, 45, 46], whereas obesity, an independent risk factor for cardiovascular disease [47], and abnormal BMI will also lead to an increased risk of developing all three complications. Elevated lipid levels, including TC and TG, are risk factors for the development of lower-limb peripheral artery disease (PAD) [48, 49] and lower extremity vascular disease, including PAD. In addition, some metabolic factors, such as TC, TG, and DBP, have been associated with the development and progression of diabetic foot and diabetic retinopathy [46, 50, 51]. Matsushita et al. showed that some kidney diseases are risk factors for PAD, diabetic foot, and diabetic retinopathy [52–54], whereas BUN is commonly used indicators to evaluate kidney function. Some studies have also suggested that elevated obesity, abnormal blood pressure, GLU2H, TC, TG and BUN levels are among the risk factors for T2DM [55–58], further validating the six risk factors as predictors of high-risk combined complications screened in this study.

### Application of a prediction model in community chronic disease management

As the frontier and important occasion for the prevention and control of DM and other chronic diseases, the community plays the role of primary (etiological prevention) and secondary (early detection, diagnosis, and treatment) in the three-level prevention strategy of chronic diseases. The high-risk complications and risk factors of T2DM screened in this study can, to a certain extent, compensate for the study gap in community T2DM management(Complication management). Simultaneously, the risk prediction model can provide medical workers with the risk of future illness based on the current health status of residents, which provides a simple and intuitive scientific tool for community T2DM complications management. First, for T2DM patients with complications, regular monitoring and health management of lower extremity vascular disease, diabetic foot, and diabetic retinopathy should be strengthened. Second, considering the cost-benefit principle, the health management of patients with T2DM is mainly focused on prevention. Intervention measures include the establishment of personal dynamic digital files of community residents (including T2DM patients with complications and high-risk groups screened by the prediction model), focusing on the risk indicators of T2DM complications and the changes in personal disease risk, formulating and adjusting personalized management plans, and strengthening health education for patients with T2DM (Especially for patients with complications), high-risk groups, and their families. Finally, this study model can assess the current community health needs by predicting the disease risk of community residents, help community managers reasonably allocate medical resources, and provide a reference for the formulation and implementation of relevant management measures.

This study has some limitations. Some data information with high missing values and unrelated indicators were selectively excluded during the data collation phase. The results may be subject to selection bias, resulting in some potential risk factors not being included in the analysis. In addition, both the training and validation sets used for modeling in this study were derived from the same data, lacking further validation in other datasets, which may affect the extrapolation ability of the model and the application effect of the model. Therefore, further prospective studies in larger populations or regions are needed to analyze and explore the validity and utility of this study’s model and related findings.

## Conclusion

In this study, a high-risk complication combination in patients with T2DM was identified among 10 common diabetic complications by association rule analysis based on the Apriori algorithm (lower extremity vascular disease, diabetic foot, and diabetic retinopathy). Divide the study population based on whether the complication combination occurred, and six risk factors (BMI, DBP, GLU2H, TC, TG and BUN) for the complication combination were screened using three methods, LASSO regression analysis, RF and SVM and established predictive models. The model performance were evaluated by using ROC curves, calibration curves and decision curves in the training and validation sets, respectively, and good performance evaluation results in all aspects.

The evaluation results showed that the model performed well in this study with high predictive power. Therefore, this model could be a useful tool for community health workers to predict the risk of high-risk complications in T2DM patients with complication and help improve the complication management of T2DM in the community. Including a focus on the development of high-risk complications such as lower extremity vascular disease, diabetic foot, and diabetic retinopathy. Through early identification of high-risk patients and timely intervention, the incidence of complications can be reduced, the quality of life of patients can be improved, and the burden on healthcare resources can be reduced. This study provides important guidance and basis for further exploration and improvement of T2DM management strategies.

## Supporting information

S1 File Original data and final analysis data used in this study. (XLSX)
